# Septins and Bacterial Infection

**DOI:** 10.3389/fcell.2016.00127

**Published:** 2016-11-11

**Authors:** Vincenzo Torraca, Serge Mostowy

**Affiliations:** Department of Medicine, MRC Centre of Molecular Bacteriology and Infection, Imperial College LondonLondon, UK

**Keywords:** actin, autophagy, cell-autonomous immunity, cytoskeleton, mitochondria, *Listeria*, *Shigella*, septins

## Abstract

Septins, a unique cytoskeletal component associated with cellular membranes, are increasingly recognized as having important roles in host defense against bacterial infection. A role for septins during invasion of *Listeria monocytogenes* into host cells was first proposed in 2002. Since then, work has shown that septins assemble in response to a wide variety of invasive bacterial pathogens, and septin assemblies can have different roles during the bacterial infection process. Here we review the interplay between septins and bacterial pathogens, highlighting septins as a structural determinant of host defense. We also discuss how investigation of septin assembly in response to bacterial infection can yield insight into basic cellular processes including phagocytosis, autophagy, and mitochondrial dynamics.

## Introduction

Work has shown that components of the cytoskeleton occupy a central role in innate immunity by promoting bacterial sensing and executing antibacterial functions (Mostowy, 2014; Mostowy and Shenoy, 2015). On the other hand, several intracellular pathogens can exploit the host cytoskeleton for their own advantage to promote invasion, establish a replicative niche, and/or enable dissemination. During infection, some bacteria can invade non-phagocytic cells including epithelial and endothelial cells (Cossart and Sansonetti, 2004; Haglund and Welch, 2011). After entry these pathogens remain enclosed within a membrane-bound compartment or escape to the host cell cytosol (Fredlund and Enninga, 2014). The compartmentalized or cytosolic lifestyle of intracellular pathogens can trigger rearrangements of the cytoskeleton and determine host response to infection. For example, some bacteria that rupture the phagocytic vacuole and escape to the cytosol directly interact with components of the host cytoskeleton to initiate actin-based motility for cell-to-cell spread (Welch and Way, 2013). To counteract bacterial pathogenesis, the cytoskeleton can mediate a variety of cell-autonomous immune defenses, such as activation of the inflammasome (a molecular platform processing inflammatory cytokines) or targeting pathogens to autophagy (a cytosolic degradation process) (Mostowy and Shenoy, 2015).

**Figure 1:**
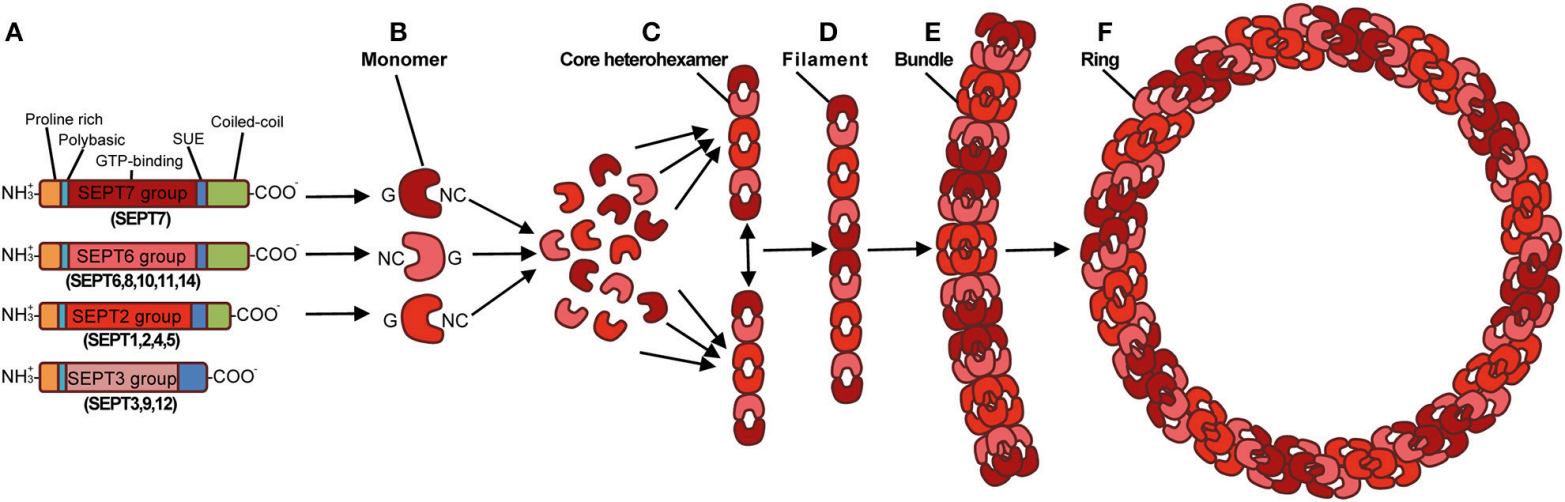
**Structure and assembly of the septin cytoskeleton. (A)** Prototypical structure of mammalian septins. Humans septins (SEPT1–SEPT12, SEPT14) are classified into four subgroups (SEPT2, SEPT3, SEPT6, SEPT7), and consist of three conserved domains: a phosphoinositide-binding polybasic region, a GTP-binding domain, and a septin unique element (SUE). Septins typically have a proline-rich motif at the amino-terminal and a coiled-coil domain at the carboxy-terminal, although the length of these regions varies. By interacting via their G and NC interfaces, septin monomers **(B)** from different subgroups (shown as different shades of red), form complexes (rods of 32–40 nm in length) that contain six **(C)** or eight (not shown) symmetrically arranged septins (2 monomers from each septin subgroup). When complexes are assembled end-to-end they form non-polar filaments **(D)**. Septin filaments can associate laterally and form bundles **(E)**, bundles of septin filaments can form higher-order structures, such as rings which are ~0.6 μm in diameter **(F)**. Adapted from Mostowy and Cossart (2012).

In comparison to actin, relatively little is known about the role of septins during bacterial infection. Septins are GTP-binding proteins that associate with cellular membrane to form filaments and ring-like structures (Mostowy and Cossart, 2012). They also interact with actin filaments and microtubules, and are therefore considered a cytoskeletal component. Septins were discovered in *Saccharomyces cerevisiae* as crucial for cell division (Hartwell, 1971). Though septins are highly conserved in fungi and animals, their number across eukaryotic species is variable. There are 7 septins in *S. cerevisiae*, 2 in *Caenorhabditis elegans*, 5 in *Drosophila melanogaster*, at least 17 in zebrafish (*Danio rerio*), and 13 in both mice and humans (SEPT1-12 and SEPT14). Vertebrate septins can be classified into 4 subgroups, namely the SEPT2, SEPT3, SEPT6, and SEPT7 subgroups, based on homology of sequence and protein domains (Mostowy and Cossart, 2012) (Figure 1A). Septin subunits from the different subgroups interact through their G (consisting of the GTP-binding domain) and NC (consisting of the amino- and carboxy-terminal regions) interfaces, forming complexes that can join end-to-end to form filaments. Septin filaments can associate, bundle, and go on to form higher-order structures such as rings (Mostowy and Cossart, 2012) (Figures 1B–F).

Septins are involved in numerous biological processes and have been implicated in a wide variety of pathological conditions including cancer, neurodegenerative disorders, and infection. This protein family was first studied in the context of bacterial infection 15 years ago (Pizarro-Cerdá et al., 2002). Identified by mass spectrometry, SEPT9 was associated with the invasion of *Listeria monocytogenes* into epithelial cells. Since then, septins have been associated with a variety of bacterial pathogens and different stages of the host cell infection process (Mostowy and Cossart, 2012; Krokowski and Mostowy, 2016). Here, we review the literature implicating septins in bacterial pathogenesis (Supplementary Table S1), and discuss how investigation of septin-bacteria interplay can provide novel insights into fundamental processes underlying bacterial infection and also host cell physiology.

## Septin function in bacterial adhesion and entry into host cells

Bacterial adhesion to the host cell is fundamental for some pathogens to establish infection. Adhesion prevents the mechanical clearance of extracellular bacteria (e.g., enteropathogenic *Escherichia coli* and *Clostridium difficile)* and facilitates the host cell entry of invasive bacteria (e.g., *L. monocytogenes, Yersinia* spp., *Shigella flexneri*, and *Salmonella* spp.). In this section, we summarize how several bacterial pathogens manipulate septins at the plasma membrane to support bacterial adherence to the host cell and enable bacterial internalization into both immune and non-immune cells.

### Formation of pedestal-like structures during enteropathogenic *Escherichia coli* infection

Enteropathogenic *E. coli* (EPEC) is a Gram-negative bacterium responsible for diarrheal disease in humans. By using a type III secretion system (T3SS), a molecular syringe that secretes effector proteins into the host cell, EPEC injects Translocated Intimin receptor (Tir) into the plasma membrane so that it functions as an anchor for Intimin, an effector protein located on the EPEC outer membrane (Croxen et al., 2013). Tir-Intimin interactions mediate the recruitment of Neural Wiskott-Aldrich Syndrome Protein (N-WASP) and the Actin-Related Protein 2/3 (ARP2/3) complex, remodeling of cortical actin filaments, flattening of intestinal microvilli, and ultimately the formation of pedestal-like structures raising the bacteria above the plasma membrane (Croxen et al., 2013; Lai et al., 2013).

**Figure 2:**
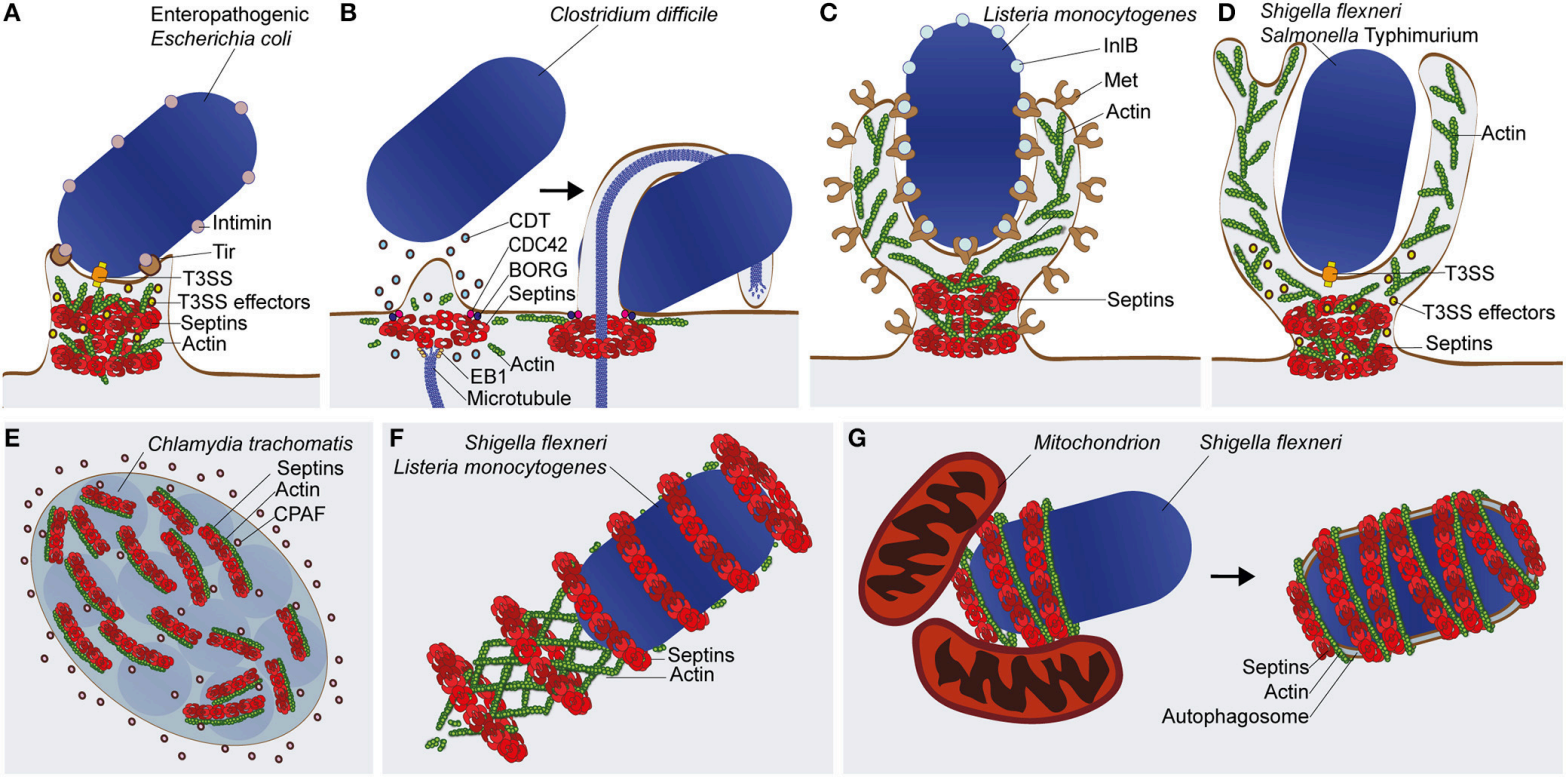
**Septin recruitment and function in bacterial infections**. **(A)** The enteropathogenic *E. coli* (EPEC) pedestal. Type 3 secretion system (T3SS) effectors of EPEC are implicated in septin phosphorylation and assembly into filaments and rings. It is proposed that septins assemblies (depicted here as rings) remodel cortical actin for pedestal biogenesis **(B)** The *C. difficile* microtubule-based protrusion. Intoxication with the *C. difficile* toxin (CDT) leads to patches of cortical actin/septin depolymerization followed by rearrangement of septins into ring-like structures, facilitated by the factors Cell Division Control Protein 42 (CDC42) and Binder of Rho GTPase (BORG). By interacting with End Binding 1 (EB1) protein, septins redirect microtubule polymerization and initiate protrusion formation. Protrusions prevent the mechanical clearance of *C. difficile* by wrapping around the bacterium. **(C)** Zipper-mediated entry by *L. monocytogenes*. During invasion of *Listeria* into host cells, interaction of the virulence factor Internalin B (InlB) with its membrane receptor Met, initiates septin recruitment to the entry site where it forms ring-like structures at the base of the phagocytic cup. **(D)** Trigger-mediated entry by *S. flexneri* or *S*. Typhimurium. Septin ring-like structures associate with the bacteria upon injection of T3SS effectors into the host cell to induce membrane ruffles and macropinocytosis at the bacterial entry site. In the case of **(C,D)**, septins at the plasma membrane may facilitate actin remodeling, membrane protrusion, and bacterial internalization by acting as diffusion barriers for lipids and signaling molecules. **(E)** The *C. trachomatis* inclusion vacuole. Septin and actin filaments form a coat that associate with the cytosolic surface of the inclusion vacuole, and contributes to its remodeling to accommodate bacterial growth. The chlamydial enzyme CPAF is able to cleave septins for control of inclusion remodeling. **(F)** The *L. monocytogenes* or *S. flexneri* actin tail. Although dispensable for actin-based motility, septins assemble into rings around a subset of cytosolic bacteria polymerizing actin tails. The precise role of septins in the functionality of the actin tail remains unknown. **(G)** The *S. flexneri* septin cage. Septins assemble into cage-like structures around a subset of cytosolic bacteria polymerizing actin. In the case of *Shigella*, septin cage assembly is promoted by mitochondria and leads to the restriction of bacterial replication by autophagy.

A recent study discovered that EPEC infection phosphorylates SEPT9 in a T3SS-dependent manner (Scholz et al., 2015). The depletion or impaired phosphorylation of SEPT9 reduced adherence of EPEC to the host cells and also EPEC-mediated cytotoxicity. How septins and their phosphorylation contribute to EPEC adhesion remains to be established. Work has shown an important role for septin phosphorylation in higher-order assembly into rings (Dobbelaere et al., 2003; Kinoshita, 2003; Hernández-Rodríguez and Momany, 2012; Meseroll et al., 2013). Given that septins are involved in actin dynamics, septin assembly may promote actin rearrangements and membrane dynamics which mediate pedestal biogenesis (Scholz et al., 2015) (Figure 2A). The T3SS effector that mediates SEPT9 phosphorylation remains unknown, however a previous study performed in *S. cerevisiae* showed that overexpression of the T3SS effector proteins Mitochondrial associated protein (Map) and *E. coli* secretion protein F (EspF) cause abnormal and mislocalized septin assemblies that affect morphogenesis and cell division (Rodríguez-Escudero et al., 2005). The same study implicated Map and EspF in the activation of a phosphorylation cascade via the Mitogen-Activated Protein Kinase (MAPK) pathway. Taken together, it is tempting to speculate that EPEC virulence factors activate a phosphorylation cascade to mediate the recruitment and assembly of septins at the plasma membrane for actin-mediated pedestal formation. Further experiments will be required to address this hypothesis. Notably, increased phosphorylation of SEPT9 has also been identified from infections with other T3SS-positive enterobacteria, including *Shigella* and *Salmonella* spp. (Rogers et al., 2011; Schmutz et al., 2013), raising the possibility that septin phosphorylation is commonly exploited by bacterial pathogens to coordinate rearrangements of the actin cytoskeleton and plasma membrane.

### Microtubule-based protrusions from *Clostridium difficile* infection

Hypervirulent strains of *C. difficile* infect humans provoking colitis, antibiotic-associated diarrhea, and occasionally death. The increased virulence of some *C. difficile* isolates is associated with the expression of the *C. difficile* transferase (CDT), a toxin that mediates formation of microtubule-based protrusions, which wrap around bacteria and increase their adherence to the host cell (Schwan et al., 2014). When exposing cells to CDT, the cellular cortex undergoes extensive remodeling, resulting in patches of actin depolymerization. Complexes of SEPT2-SEPT6-SEPT7-SEPT9 are recruited to the plasma membrane shortly after initial depolymerization events and form collar-like structures that initiate CDT-dependent protrusions (Nölke et al., 2016) (Figure 2B). Depletion of septins by siRNA or their inhibition with forchlorfenuron reduced protrusion formation, while septin overexpression increased protrusion emergence. At the protrusion base, septins interact with the GTPase Cell Division Control Protein 42 (CDC42) and its effector Binder of Rho GTPase (BORG) (Nölke et al., 2016). These results are consistent with previous work using non-infected HeLa cells showing the regulation of septin assembly by BORGs (Joberty et al., 2001).

Septins have high affinity to End Binding 1 (EB1), a protein which associates to the plus end of microtubules, suggesting that septins guide microtubule polymerization in the extending protrusion (Nölke et al., 2016). The discovery that septins mediate microtubule apical guidance can have broad implications in cell biology. Septins are well known to localize at the base of the primary cilium, a membrane-delimited eukaryotic appendage also sustained by a microtubule-based scaffold that senses and transduces a variety of extracellular stimuli (Hu et al., 2010). Work has shown that depletion of SEPT2 results in mislocalization of ciliary membrane proteins and impairs ciliogenesis (Hu et al., 2010). Together, these results indicate that septins play a key role in maintenance of membrane compartmentalization and work as a membrane diffusion barrier, a function also attributed to septin rings at the mother-bud neck in *S. cerevisiae* (Barral et al., 2000; Takizawa et al., 2000; Caudron and Barral, 2009).

### “Zipper” and “trigger”-mediated entry of invasive bacteria

To enter non-phagocytic cells, invasive bacteria have different mechanisms to manipulate host signaling pathways leading to their uptake. Some bacteria, such as *L. monocytogenes* and *Yersinia* spp., can enter host cells via a mechanism called “zippering” that is activated via the direct interaction between bacterial surface components and host cell receptors at the plasma membrane. Other bacteria, such as *S. flexneri* and *Salmonella enterica* serovar Typhimurium, enter non-phagocytic cells by a mechanism called “triggering” that is dependent on the injection of T3SS effector proteins to stimulate host cell membrane ruffling and engulfment via a macropinocytosis-like process.

*L. monocytogenes*, a Gram-positive bacterium and foodborne pathogen, can stimulate its internalization into host cells via interactions between its surface proteins Internalin (InlA) and Internalin B (InlB) with plasma membrane proteins E-cadherin and Met, respectively (Pizarro-Cerdá et al., 2012). To discover other proteins required for *Listeria* entry, proteomic analysis was performed and identified enrichment of SEPT9 at the entry site during InlB-mediated invasion (Pizarro-Cerdá et al., 2002). Follow-up work revealed that septin collar-like structures assembled in response to actin polymerization at sites of *Listeria* entry (Mostowy et al., 2009b) (Figure 2C). Interestingly, SEPT2 promotes *Listeria* invasion while SEPT11 restricts it, suggesting different roles for different septins in bacterial entry (Mostowy et al., 2009a,b, 2011a; Kühbacher et al., 2015). Septins also form collar-like structures at sites of entry of “triggering” bacteria, such as *S. flexneri* and *S*. Typhimurium (Mostowy et al., 2009b) (Figure 2D). Here, septins may additionally facilitate membrane fusion events during the bacteria-induced macropinosome trafficking (Dolat and Spiliotis, 2016; Weiner et al., 2016). Together, this highlights septin assembly as a general response to actin-mediated bacterial invasion. In addition to the above studies performed using infection of non-phagocytic host cells, work has shown that SEPT2 and SEPT11 assemble into collar-like structures at the base of the phagocytic cup in macrophages and neutrophils to enable phagocytosis (Huang et al., 2008).

The precise role of septins in mediating bacterial entry remains to be established. Considering their function in compartmentalizing plasma membrane (Kusumi et al., 2012; Bridges and Gladfelter, 2015), septins may recruit host cell receptors, phospholipids, and/or signaling molecules to orchestrate the cytoskeletal rearrangements and membrane extension which underpin formation of the phagocytic cup (Barral et al., 2000; Caudron and Barral, 2009; Mostowy and Cossart, 2011; Ostrowski et al., 2016).

## Septin interactions with intracellular bacterial pathogens

After phagocytosis and entry into host cells, bacteria can adopt different intracellular lifestyles. Some invasive pathogens, such as *Chlamydia trachomatis* and *Legionella pneumophila*, can remodel their phagocytic compartment to establish an intracellular niche for persistence and replication. In contrast, other bacterial pathogens such as *L. monocytogenes, S. flexneri, Mycobacterium marinum*, and *Rickettsia* spp., can escape from the phagocytic vacuole to invade the host cell cytosol (Ray et al., 2009). In the following section, we discuss the interactions between septins and intracellular bacteria. We focus on septin roles in maintenance of the *C. trachomatis* intracellular niche, and also in the compartmentalization of cytosolic *S. flexneri* for host defense.

### Septin interactions with *Chlamydia trachomatis*

*C. trachomatis* is an obligate intracellular bacterium that replicates inside an inclusion vacuole delimited by host membranes. The parasitic lifecycle of *Chlamydia* relies on the recruitment of actin and vimentin intermediate filaments to the inclusion (Kumar and Valdivia, 2008). To remodel the cytoskeleton, *C. trachomatis* secretes the protease Chlamydial proteasome-like activity factor (CPAF) into the host cell cytosol and increases the size/flexibility of the inclusion to enable bacterial replication (Kumar and Valdivia, 2008). A recent study revealed that complexes of SEPT2-SEPT11-SEPT7-SEPT9 are also recruited to the cytosolic surface of the *Chlamydia* inclusion, where septins associate with actin filaments (Volceanov et al., 2014) (Figure 2E). Inhibition of septin assembly using forchlorfenuron reduced the expansion of inclusion vacuoles. Additionally, depletion of septins by siRNA abolished the formation of septin/actin coats around the *Chlamydia* inclusion and restricted the extrusion of intact inclusions from host cells. The same study revealed that SEPT2 is a host cell substrate for CPAF, like vimentin. While the exact role of SEPT2-CPAF interactions awaits investigation, it has been suggested that cleavage of SEPT2 by CPAF contributes to dynamic remodeling of the cytoskeleton around the inclusion.

### A role for septins in actin tail polymerization?

To evade cytosolic immunity, some bacteria that escape from the phagocytic vacuole can polymerize actin for actin-based motility and cell-to-cell dissemination (Welch and Way, 2013). Interestingly, bacteria (including *Listeria* spp., *Shigella* spp., *M. marinum, Burkholderia* spp., and *Rickettsia* spp.) have evolved different mechanisms to polymerize actin tails (Gouin et al., 2005; Welch and Way, 2013). Just as septins are recruited to actin polymerization at the plasma membrane, septins are also recruited to some actin-polymerizing bacteria in the cytosol as ring-like structures (Mostowy et al., 2010) (Figure 2F). Although work using septin-depleted cells failed to reveal any role for septins in the functionality of actin tails (Mostowy et al., 2010), it is possible that septins impact actin tails in aspects difficult to detect using transient depletion techniques in tissue culture cells. Given that both actin-based motility and septin assembly can be reconstituted using purified proteins in cell-free conditions (Loisel et al., 1999; Bridges and Gladfelter, 2016), it seems likely that biochemical reconstitution systems will be of great value to elucidate whether septins have a role in the functionality of actin tails.

### Septin interplay with bacterial autophagy

Septins can recognize some bacterial pathogens that escape to the host cell cytosol and polymerize actin, forming cage-like structures to entrap bacteria and prevent actin tail formation (Mostowy et al., 2010) (Figure 2G). Indeed, the frequency of actin tails is significantly enhanced in septin-depleted cells infected with bacteria normally recognized by septin cages. Recent work has also shown that septin cages assemble to restrict bacterial replication (Sirianni et al., 2016). Septin cages have been observed for both *S. flexneri* and *M. marinum* during infection of tissue culture cells *in vitro* and during infection of zebrafish *in vivo* (Mostowy et al., 2010, 2013; Sirianni et al., 2016). Collectively, these observations highlight septin caging of cytosolic bacteria as an evolutionarily-conserved assembly valuable for host defense (Mostowy, 2014; Mostowy and Shenoy, 2015).

*Shigella* entrapped in septin cages are also targeted to autophagy (Mostowy et al., 2010; Sirianni et al., 2016) (Figure 2G). Autophagy is a membrane trafficking process that can redirect cytosolic material, including bacteria, to lytic compartments (Levine et al., 2011; Mostowy, 2014). A group of autophagy-related (ATG) proteins constitute the core machinery responsible for the formation, elongation, and maturation of the autophagosome. In the case of bacterial autophagy, ubiquitination and its recognition by a set of ubiquitin-binding autophagy receptors (e.g., p62/SQSTM1, NDP52/CALCOCO2), can direct the ATG machinery around bacterial cargo (Khaminets et al., 2016; Maculins et al., 2016). Strikingly, the recruitment of septins and autophagy markers are tightly connected. The depletion of septins (SEPT2, SEPT7, or SEPT9) or autophagy components (p62, NDP52, ATG5, ATG6, or ATG7) abrogates both septin cage assembly and bacterial autophagy (Mostowy et al., 2010, 2011b; Sirianni et al., 2016). These data suggest that, at least during autophagy of *Shigella*, septin cage assembly and autophagosome formation are interdependent (Mostowy and Cossart, 2012). It is interesting to consider that septin cages have been observed during autophagy of *S. flexneri* and *M. marinum*, distinct bacterial pathogens that both polymerize actin via the recruitment of N-WASP (Mostowy et al., 2010, 2013; van der Vaart et al., 2014). In contrast, septin cages have not been observed for *Listeria* spp. (Mostowy et al., 2010), partly because cytosolic *Listeria* polymerize actin tails via ActA, a bacterial effector that mimics host cell N-WASP and prevents the ubiquitination and recognition of bacteria by autophagy (Yoshikawa et al., 2009; Mostowy et al., 2011b). How exactly septins and autophagy are recruited to bacteria remains to be established. Recent work in *S. cerevisiae* has shown that septins are involved in the early stages of autophagosome formation during starvation (Barve et al., 2016). Collectively, these data suggest that septins have a fundamental role in multiple pathways of autophagy, including starvation-induced autophagy and bacterial autophagy.

To better understand the molecules and events required for septin cage assembly, proteomic analysis of the *Shigella*-septin cage was performed (Sirianni et al., 2016). From this approach, 56 host proteins were identified to interact with septins in *Shigella*-infected conditions, including p62 and the ATG8 family member Light Chain 3 B (LC3B). Surprisingly, proteomics also revealed that 21.4% of septin cage-associated proteins were annotated as exclusively mitochondrial. Indeed, high-resolution microscopy showed that mitochondria support septin assembly into the cages that entrap *Shigella* for autophagy. A role for mitochondria in septin cage assembly was confirmed by depletion of factors implicated in mitochondria fission and fusion (Sirianni et al., 2016). The depletion of Dynamin Related Protein 1 (DRP1), which elongates mitochondria and increases their availability as a membrane source for septin assembly, significantly increased septin caging. Conversely, the depletion of Mitofusin 1 (MFN1), which fragments mitochondria and limits their availability as a membrane source for septin assembly, significantly reduced septin caging. *Shigella* invasion is well known to induce mitochondria fragmentation (Carneiro et al., 2009; Lum and Morona, 2014). Remarkably, fragmentation of mitochondria enables *Shigella* to escape from septin cages and autophagy recognition (Sirianni et al., 2016). *L. monocytogenes*, which avoids septin caging, can induce fragmentation of mitochondria via Listeriolysin O (LLO), a pore-forming toxin (Stavru et al., 2011). Together, these observations suggest that fragmentation of mitochondria may be a general mechanism for bacterial pathogens to escape from cell-autonomous immunity (Sirianni et al., 2016).

Important studies have shown that septins recognize micron scale curvature at the plasma membrane, and that septin assembly is membrane-facilitated (Tanaka-Takiguchi et al., 2009; Bridges and Gladfelter, 2015; Bridges et al., 2016). In agreement with this, septin assemblies are closely associated with invaginations and protrusions of the plasma membrane including the phagocytic cup, the cleavage furrow, and the base of dendritic spines and cilia (Bridges et al., 2016; Lobato-Márquez and Mostowy, 2016). In contrast, septin association with sources of membrane in the cytosol remains to be established. New studies have shown that septins interact with mitochondria and enable mitochondrial fission, in a process called mitokinesis (Pagliuso et al., 2016; Sirianni et al., 2016). Strikingly, septin function at the plasma membrane (e.g., phagocytosis, cytokinesis) and in the cytosol (e.g., septin caging, mitokinesis) all involve actin and non-muscle myosin II (Mostowy and Cossart, 2012; Sirianni et al., 2016). Next, it will be important to investigate different sources of cytosolic membrane (e.g., mitochondria, ER), and their precise role in septin assembly.

## Conclusions

Investigation into the cell biology of bacterial infection has significantly improved our understanding of both infection and cytoskeleton biology (Haglund and Welch, 2011). For example, the study of actin-tail formation by cytosolic bacteria has enabled major discoveries underlying bacterial pathogenesis and actin biology (Welch and Way, 2013). In this review, we have discussed several examples of how investigation of septin assembly in response to bacterial invasion is discovering novel aspects of septin biology and cell-autonomous immunity. Our current knowledge of septin function during infection mostly derives from work performed *in vitro* using non-phagocytic cells (e.g., HeLa). Considering the emerging roles for the host cytoskeleton in cell-autonomous immunity (Mostowy and Shenoy, 2015), future work should address septin function in immune cells. More insight into septin roles in host defense can also come from the use of animal models. Indeed, septin-bacteria interactions can be studied using zebrafish infection models innovated to study the cell biology of infection *in vivo* (Mostowy et al., 2013; Willis et al., 2016). We propose that use of *in vivo* systems, including the zebrafish and mouse, will be essential to illuminate a more complete understanding of septin biology during host defense against bacterial infection.

## Author contributions

VT and SM jointly wrote the manuscript.

### Conflict of interest statement

The authors declare that the research was conducted in the absence of any commercial or financial relationships that could be construed as a potential conflict of interest.
