# Supplementary material for: Septins and Bacterial Infection
Source: Front Cell Dev Biol. 2016 Nov 11;4:127. doi: 10.3389/fcell.2016.00127 (PMC5104955; doi:10.3389/fcell.2016.00127)
Supplement: Supplementary file 1 [file DataSheet1.DOCX]

**Supplementary Table S1. Septin interactions with different bacterial pathogens.**

| **Adhesion of extracellular pathogens to the host cell** | | | | |
| --- | --- | --- | --- | --- |
| **Structure** | **Role of septins** | **Pathogen** | | **Reference** |
| Pedestal | Remodeling of cortical actin for pedestal biogenesis | Enteropathogenic *Escherichia coli* (EPEC) | | (Scholz et al., 2015) |
| Microtubule-based protrusion | Redirection of polymerizing microtubules to protrusion site | *Clostridium difficile* | | (Nolke et al., 2016) |
| **Bacterial entry** | | | | |
| **Structure** | **Role of septins** | **Pathogen** | | **Reference** |
| Phagocytic cup | Invasion of bacteria via the “zipper entry” mechanism | *Listeria monocytogenes* | | (Pizarro-Cerda et al., 2002;Mostowy et al., 2009) |
| Membrane ruffle, Macropinocytosis | Invasion of bacteria via the “trigger entry” mechanism | *Shigella flexneri*  *Salmonella enterica* Serovar Typhimurium | | (Mostowy et al., 2009) |
| **Interplay with compartimentalized bacteria** | | | | |
| **Structure** | **Role of septins** | **Pathogen** | | **Reference** |
| Inclusion vacuole | Stabilization of actin coats around inclusion vacuoles and extrusion of intact inclusions | | *Chlamydia trachomatis* | (Volceanov et al., 2014) |
| **Interplay with cytosolic bacteria** | | | | |
| **Structure** | **Role of septins** | **Pathogen** | | **Reference** |
| Actin tail | Unknown, dispensable for actin based motility | | *Listeria monocytogenes*  *Shigella flexneri* | (Mostowy et al., 2010) |
| Septin cage | Restriction of bacterial replication and cell-to-cell dissemination, targeting of bacteria to autophagy | | *Shigella flexneri*  *Mycobacterium marinum* | (Mostowy et al., 2010;Mostowy et al., 2013;Sirianni et al., 2016) |

**Supplemental Table S1 References**

Mostowy, S., Bonazzi, M., Hamon, M.A., Tham, T.N., Mallet, A., Lelek, M., Gouin, E., Demangel, C., Brosch, R., Zimmer, C., Sartori, A., Kinoshita, M., Lecuit, M., and Cossart, P. (2010). Entrapment of intracytosolic bacteria by septin cage-like structures. *Cell Host Microbe* 8**,** 433-444.

Mostowy, S., Boucontet, L., Mazon Moya, M.J., Sirianni, A., Boudinot, P., Hollinshead, M., Cossart, P., Herbomel, P., Levraud, J.P., and Colucci-Guyon, E. (2013). The zebrafish as a new model for the in vivo study of Shigella flexneri interaction with phagocytes and bacterial autophagy. *PLoS Pathog* 9**,** e1003588.

Mostowy, S., Nam Tham, T., Danckaert, A., Guadagnini, S., Boisson-Dupuis, S., Pizarro-Cerda, J., and Cossart, P. (2009). Septins regulate bacterial entry into host cells. *PLoS One* 4**,** e4196.

Nolke, T., Schwan, C., Lehmann, F., Ostevold, K., Pertz, O., and Aktories, K. (2016). Septins guide microtubule protrusions induced by actin-depolymerizing toxins like Clostridium difficile transferase (CDT). *Proc Natl Acad Sci U S A* 113**,** 7870-7875.

Pizarro-Cerda, J., Jonquieres, R., Gouin, E., Vandekerckhove, J., Garin, J., and Cossart, P. (2002). Distinct protein patterns associated with Listeria monocytogenes InlA- or InlB-phagosomes. *Cell Microbiol* 4**,** 101-115.

Scholz, R., Imami, K., Scott, N.E., Trimble, W.S., Foster, L.J., and Finlay, B.B. (2015). Novel Host Proteins and Signaling Pathways in Enteropathogenic E. coli Pathogenesis Identified by Global Phosphoproteome Analysis. *Mol Cell Proteomics* 14**,** 1927-1945.

Sirianni, A., Krokowski, S., Lobato-Marquez, D., Buranyi, S., Pfanzelter, J., Galea, D., Willis, A., Culley, S., Henriques, R., Larrouy-Maumus, G., Hollinshead, M., Sancho-Shimizu, V., Way, M., and Mostowy, S. (2016). Mitochondria mediate septin cage assembly to promote autophagy of Shigella. *EMBO Rep* 17**,** 1029-1043.

Volceanov, L., Herbst, K., Biniossek, M., Schilling, O., Haller, D., Nolke, T., Subbarayal, P., Rudel, T., Zieger, B., and Hacker, G. (2014). Septins arrange F-actin-containing fibers on the Chlamydia trachomatis inclusion and are required for normal release of the inclusion by extrusion. *MBio* 5**,** e01802-01814.
